# Delivery of miR-214 via extracellular vesicles downregulates *Xbp1* expression and pro-inflammatory cytokine genes in macrophages

**DOI:** 10.20517/evcna.2023.64

**Published:** 2024-05-28

**Authors:** Gonzalo Almanza, Stephen Searles, Maurizio Zanetti

**Affiliations:** The Laboratory of Immunology, Department of Medicine and Moores Cancer Center, University of California, San Diego, La Jolla, CA 92093, USA.

**Keywords:** Tumor microenvironment, UPR, XBP1, macrophages, extracellular vesicles, miR-214

## Abstract

**Aim:**

Tumor-infiltrating macrophages are tumor-promoting and show activation of the unfolded protein response (UPR). The transcription factor X-box binding protein 1 (XBP1) is a conserved element of the UPR. Upon activation, the UPR mediates the transcriptional activation of pro-inflammatory cytokines and immune suppressive factors, hence contributing to immune dysregulation in the tumor microenvironment (TME). miR-214 is a short non-coding miRNA that targets the 3’-UTR of the Xbp1 transcript. Here, we tested a new method to efficiently deliver miR-214 to macrophages as a potential new therapeutic approach.

**Methods:**

We generated miR-214-laden extracellular vesicles (iEV-214) in a murine B cell and demonstrated that iEV-214 were enriched in miR-214 between 1,500 – 2,000 fold relative to control iEVs.

**Results:**

Bone marrow-derived macrophages (BMDM) treated with iEV-214 for 24 h underwent a specific enrichment in miR-214, suggesting transfer of the miR-214 payload from the iEVs to macrophages. iEV-214 treatment of BMDM markedly reduced (> 50%) Xbp1 transcription under endoplasmic reticulum stress conditions compared to controls. Immune-related genes downstream of XBP1s (*Il-6*, *Il-23p19*, and *Arg1*) were also reduced by 69%, 51%, and 34%, respectively.

**Conclusions:**

Together, these data permit to conclude that iEV-214 are an efficient strategy to downregulate the expression of *Xbp1* mRNA and downstream genes in macrophages. We propose miRNA-laden iEVs are a new approach to target macrophages and control immune dysregulation in the TME.

## INTRODUCTION

Mammalian cells cope with endoplasmic reticulum (ER) stress by initiating the unfolded protein response (UPR), a homeostatic mechanism operational in yeasts, mammals, and plants^[1]^. This response is mediated by three initiator/sensor molecules: inositol-requiring enzyme 1 (IRE1α), PKR-like ER kinase (PERK), and activating transcription factor 6 (ATF6), which are maintained in an inactive state through association with 78 kDa glucose-regulated protein (GRP78)^[2]^. Upon ER stress, GRP78 disassociates from the three sensors, allowing their activation and initiating downstream signaling. During this process, the endoribonuclease IRE1α initiates the unconventional splicing of the mRNA encoding X-box binding protein 1 (XBP1). Spliced XBP1 (XBP-1s) is a potent transcriptional activator that increases the expression of a subset of UPR-related genes involved in protein folding, maturation, and degradation in the ER^[3]^. The IRE1-XBP1 axis is phylogenetically conserved and exerts functions related to the biology of immune cells. In the B cell lineage, XBP1 is required for the terminal differentiation into plasma cells^[4]^. In activated T cells, XBP1 is activated and remains in a state of activation for several days, possibly to compensate for the synthesis of cytokines^[5,6]^. In dendritic cells, XBP1 is essential in development and survival^[7]^ but also in the control of prostaglandin biosynthesis and pain mediation^[8]^. In macrophages, the IRE1-XBP1 axis plays a role in polarization to a mixed pro-inflammatory/ immune suppressive phenotype that is associated with tumor progression^[9,10]^.

Cancer cells of various origins undergoing an ER stress response/UPR release diffusible factors that transmit ER stress to receiver myeloid cells, macrophages and dendritic cells, eliciting the transcription of pro-inflammatory/tumorigenic cytokines such as IL-6 and IL-23 and immune suppressive factors such as Arginase1 (Arg1)^[6,9]^. It has been demonstrated that the promoter region of these pro-inflammatory cytokines possesses binding sites for the spliced isoform of XBP1^[11]^. Thus, a UPR-based inter-cellular communication links together tumor cells and myeloid cells, driving their polarization in the TME^[12]^.

Recently, we demonstrated that conditional deletion of genes in the IRE1-XBP1 branch of the ER stress pathway (*Ern1*and *Xbp1*, respectively) renders macrophages resistant to the negative effects of transmissible (cell nonautonomous) ER stress^[10]^. Furthermore, mice with a conditional deletion of the *Ern1* gene that are inoculated with B16 melanoma cells survive significantly longer than wild-type mice^[10]^. This showed that targeting the IRE1-XBP1 axis could be a novel strategy to regulate myeloid cells in the TME. There are no FDA-approved small molecule inhibitors of IRE1 or XBP1, but a systemic administration would be fraught with considerable off-target effects in normal cells. Therefore, new methods to target macrophages and regulate the IRE1-XBP1 pathway are needed.

In eukaryotes, evolutionarily conserved 20–30 nucleotides non-coding RNAs (miRNAs) regulate gene expression by binding to sequences with partial complementarity at the 3’-UTR (rarely the 5’-UTR) of target RNA transcripts, causing messenger RNA degradation and/or post-transcriptional regulation of gene expression^[13]^. miRNAs are involved in the regulation of a variety of cellular processes, including cancer cell growth and metastasis^[14–17]^. Recently, this laboratory developed a novel technology wherein B lymphocytes can be programmed with suitable engineered plasmid DNA to enforce the release of extracellular vesicles (iEVs) with a predetermined miRNA payload^[18,19]^.

Regulation of the IRE1-XBP1 axis via miR-214 has been documented in both cancer and non-cancer systems^[20–22]^. Here, we examined the effects of targeting bone marrow-derived macrophages (BMDM) using miR-214-laden iEVs generated in B cells. We found that iEV-214 treatment markedly reduced (> 50%) Xbp1 transcription under endoplasmic reticulum stress conditions. This represents a first step toward new forms of therapy targeting the IRE1-XBP1 axis in macrophages in the TME to control local immune dysregulation and cancer progression.

## MATERIALS AND METHODS

### pCMV-MIR cloning and validation

A miR construct containing the precursor miR-214 hairpin plus 75 bp flanking sequences was synthesized with SgfI/ XhoI restriction sites by Integrated DNA Technologies (IDT, Coralville, IA)^[23]^. A construct containing a scrambled miRNA sequence with SgfI/ XhoI restriction sites was also generated. Each construct was cloned into the pCMV-MIR expression plasmid (Origene, Rockville, MD) by digestion with SgfI and XhoI and subsequent ligation. The ligation mixture was transformed into TOP10 competent cells (Life Technologies, Carlsbad, CA). After transformation, clones were selected and grown overnight at 37 °C. DNA was extracted with Promega Wizard Plus SV Minipreps DNA Purification System (Promega, Madison WI). The resulting plasmids were termed pCMV-MIR-214 and pCMV-MIR-scr. Each plasmid was further analyzed by restriction mapping with SgfI/ XhoI digest and gel electrophoresis.

### Cell culture and transfection

J558L mouse B-cell myeloma cells were grown in suspension to 80% confluence in RPMI with 10% fetal bovine serum that had been depleted of exosomes via ultracentrifugation at 100,000 × *g* for 2 h^[19]^. Cells (2 × 106) were transfected with pCMV-MIR plasmid (1 μg of either pCMV-MIR-214 or pCMV-MIR-scr) utilizing the Lonza VACA-1003 transfection kit V and Nuclefector 2b device (Lonza, Walkersville, MD)^[19]^. Cells were allowed to recover in a T25 flask upright at 37 °C with 5% CO for 48 h before iEV isolation.

### Isolation of iEVs

iEVs were isolated from cell culture per our published procedure^[19]^. Briefly, 48 h post-transfection, 5 mL of culture supernatant was collected and incubated with 5 mL of Total Exosome Isolation kit (Life Technologies, Carlsbad, CA) at room temperature for 1 h. The resulting mixture was centrifuged at 16,000 RPM at 4 °C for 1 h, and the iEV pellet was resuspended in 250 μL of PBS at room temperature and stored in 1.5 mL Eppendorf tubes at −20 °C until use. EVs isolated from J558L cells either untransfected or transfected with pCMV-MIR-scr served as controls.

### miR-specific qPCR

RNA extraction for miR-specific qPCR was performed as described^[19]^. Isolated iEVs and iEV-treated BMDM were subject to total RNA extraction using the ZYGEM RNAtissue Plus System (Zygem, Hamilton, NZ) according to the manufacturer’s instructions. For miRNA quantification, cDNA was generated from intracellular and exosome miRNA with Taqman small RNA assays^[19]^. Input RNA was normalized to 100 ng/sample for iEVs and cellular RNA. Taqman MicroRNA Reverse Transcription Kit was utilized for all samples following the manufacturer’s instructions. Cycling conditions for qPCR were: 40 cycles, 96 °C denature 30 seconds, 60 °C anneal/extension 30 seconds. SnoRNA202 (Cat. # 4398967 Life Technologies) was used for standardization. Results are displayed as fold change, calculated using the 2-ddCt method. If no Ct value was available for control conditions, raw Ct values are shown instead.

### Standard qPCR

Standard qPCR was performed as described previously^[10]^. Briefly, mRNA was harvested from cells using Nucleopsin II Kit (Machery-Nagel). The concentration and purity of RNA were quantified using the NanoDrop (ND-1000) spectrophotometer (Thermo Scientific) and analyzed with NanoDrop Software v3.8.0. RNA was normalized between conditions and cDNA generated using the High Capacity cDNA Synthesis kit (Life Technologies). RT-qPCR was performed on ABI 7300 Real-Time PCR system using TaqMan reagents for 50 cycles using universal cycling conditions. Cycling conditions followed the manufacturer’s specifications (KAPA Biosystems). Target gene expression was normalized to *β-actin* and relative expression determined by using the 2-ddCt relative quantification method. The RT-qPCR primers for *Xbp1* (Mm00457357_m1 ), *Arg1* (Mm00475988_m1), *Il6* (Mm99999064_m1), and *Il23-p19* (Mm00518984_m1) were purchased from Life Technologies. The sequence of the primers is proprietary information of Life Technologies.

### Generation of TERS

TERS (transmissible ER stress) conditioned medium (CM) was generated in colon cancer DLD1 cells treated with thapsigargin 0.3 μM (Enzo Life Sciences) for 2 h^[10]^. Cells were then washed twice with Dulbecco’s PBS (Corning) and incubated in fresh, standard growth medium for 16 additional hours. The TERS CM was harvested, centrifuged for 10 min at 2,000 RPM, filtered through a 0.22 μm filter (Millipore), and stored at −80 °C until use.

### Generation of BMDM

BMDM were isolated from the femur and tibia of wild-type C57/Bl6 mice by flushing out the bone marrow using cold, serum-free RPMI growth media (Corning) with a 27-gauge needle and syringe. Macrophage differentiation was obtained through incubation in standard growth medium supplemented with m-CSF (Peprotech) at 30 ng/mL for 7 days^[10]^. BMDM (5 × 10^5^) were treated with iEVs (10 μg/mL) in 6-well plates for 24 h. After incubation, cells were washed twice and RNA was isolated as described above. Procedures were per protocol approved by the Institutional Animal Care and Use Committee (IACUC) and in compliance with Association for Assessment Accreditation of Laboratory Animal Care (AAALAC) International guidelines.

## RESULTS

### Engineering iEV-214

**Figure 1.**
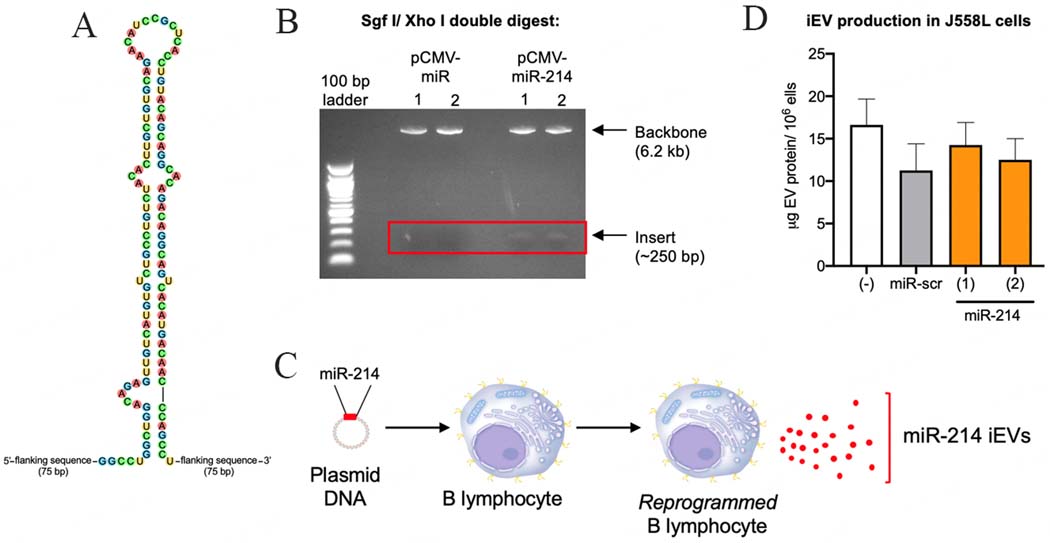
Engineering pCMV-MIR-214 and generation of iEV-214. (A) Sequence of pre-miR-214 minigene that was cloned into pCMVMIR to generate pCMV-MIR-214; (B) Restriction mapping of pCMV-MIR (empty) and pCMV-MIR-214. Plasmids were double digested with SgfI and XhoI and fragments were resolved via electrophoresis; (C) Schematic representation depicting the generation of iEV-214; (D) Quantification of iEVs purified from the conditioned media of J558L cells that were untransfected (−) or transfected with pCMV-MIR-scr or pCMV-MIR-214. iEV protein content was quantified using the BCA assay.

We cloned a minigene encoding for the miR-214 precursor hairpin (pre-miR) along with 75 bp flanking sequences into the miR expression plasmid pCMV-MIR per our previous method^[9]^ [Figure 1A]. We used restriction mapping to show that the minigene was correctly cloned into the plasmid, yielding pCMV-MIR-214 [Figure 1B]. We transfected pCMV-MIR-214 into the murine B lymphoma cell line J558L to produce and secrete iEV-214 [Figure 1C]. We observed no differences in vesicle production between untransfected and transfected (pCMV-MIR-214 or pCMV-MIR-scr) J558L cells [Figure 1D], ruling out the possibility that miR-214 might negatively impact the biosynthetic machinery. It is known that Xbp1 supports the expansion of the endoplasmic reticulum during immunoglobulin synthesis in plasma cells^[4]^.

### miR-214 is enriched in iEVs and is delivered to BMDM

**Figure 2.**
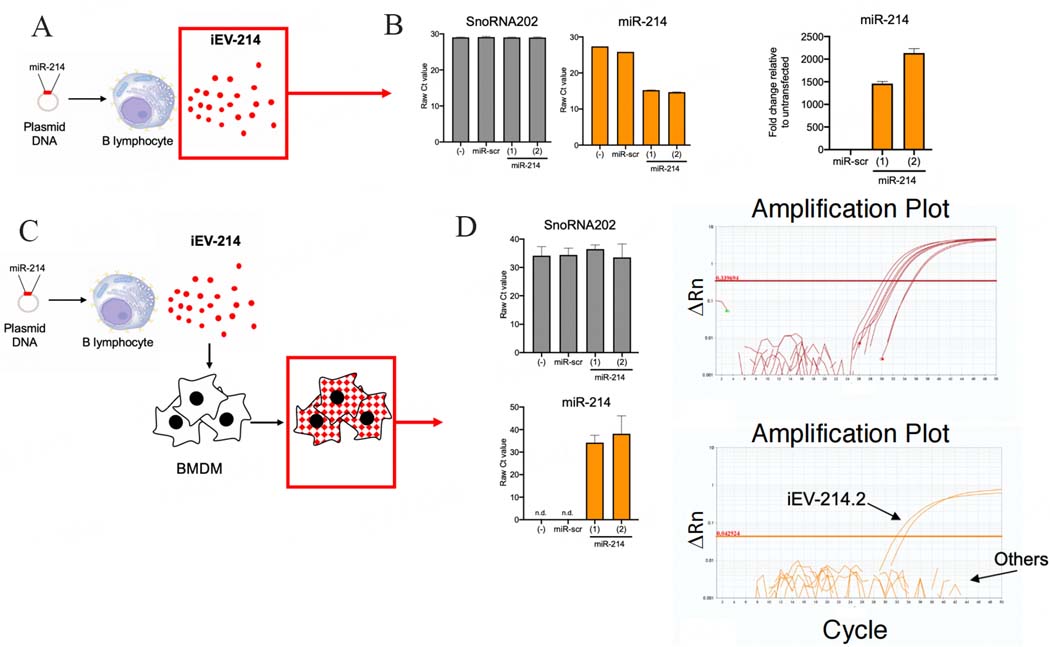
miR-214 is enriched in iEVs and can be transferred to BMDM. (A) Schematic representation depicting the biological material (iEVs) analyzed in B; (B) Bar graphs depicting: Ct values for SnoRNA202 and miR-214, and fold change in miR-214 in iEVs subject to miR-specific qPCR. Fold change was calculated relative to iEVs from untransfected J558L cells and normalized using SnoRNA202; (C) Schematic representation depicting the biological material (BMDM treated with iEVs) analyzed in D; (D) Bar graphs depicting Ct values and amplification plots for SnoRNA202 (upper) and miR-214 (lower) from BMDM treated with iEVs.

We sought to determine if iEVs are enriched in cargo miR-214. To this end, we performed miR-specific qPCR directly on iEVs purified (48 h post-transfection) from the supernatant of J558L cells transfected with pCMV-MIR-214 or pCMV-MIR-scr as control. EVs from untransfected cells were used as an additional control [Figure 2A]. We measured Ct values of miR-214 and contextually of SnoRNA202, a “housekeeping” miR known to be expressed in B cell EVs. We found that whereas the Ct values of SnoRNA202 were remarkably similar across all conditions tested (Ct = 29.0–29.3), Ct values of miR-214 were markedly lower in iEV-214 (Ct = 14.6–15.2) compared to iEV-scr (Ct = 25.8) or iEVs from untransfected J558L cells (Ct = 27.4), suggesting a dramatic enrichment of miR-214 in iEV-214. We estimated this to be in the order of 1,500–2,000-fold higher relative to control iEVs [Figure 2B].

Next, we sought transfer of miR-214 from iEV-214 to target macrophages. To this end, murine BMDM were treated with iEV-214 or control iEVs (iEV-scr and EVs from untransfected J558L cells). After 24 h, we measured the amount of miR-214 in BMDM using miR-specific qPCR [Figure 2C]. We found that whereas the Ct values for SnoRNA202 were similar across all conditions tested (Ct = 33.6–36.5), miR-214 was detected in BMDM treated with iEV-214 (Ct = 34.2–38.2) [Figure 2D], but not in any of the other control conditions, suggesting that the endogenous level of miR-214 in BMDM is below the detection limit of our assay. Collectively, these results show that B cells can be engineered to secrete iEVs with a miR-214 cargo, and that iEV-214 can transfer their miR-214 cargo to BMDM.

### Transfer of miR-214 to BMDM downregulates Xbp1 expression and reduces the pro-inflammatory/immune suppressive phenotype

**Figure 3.**
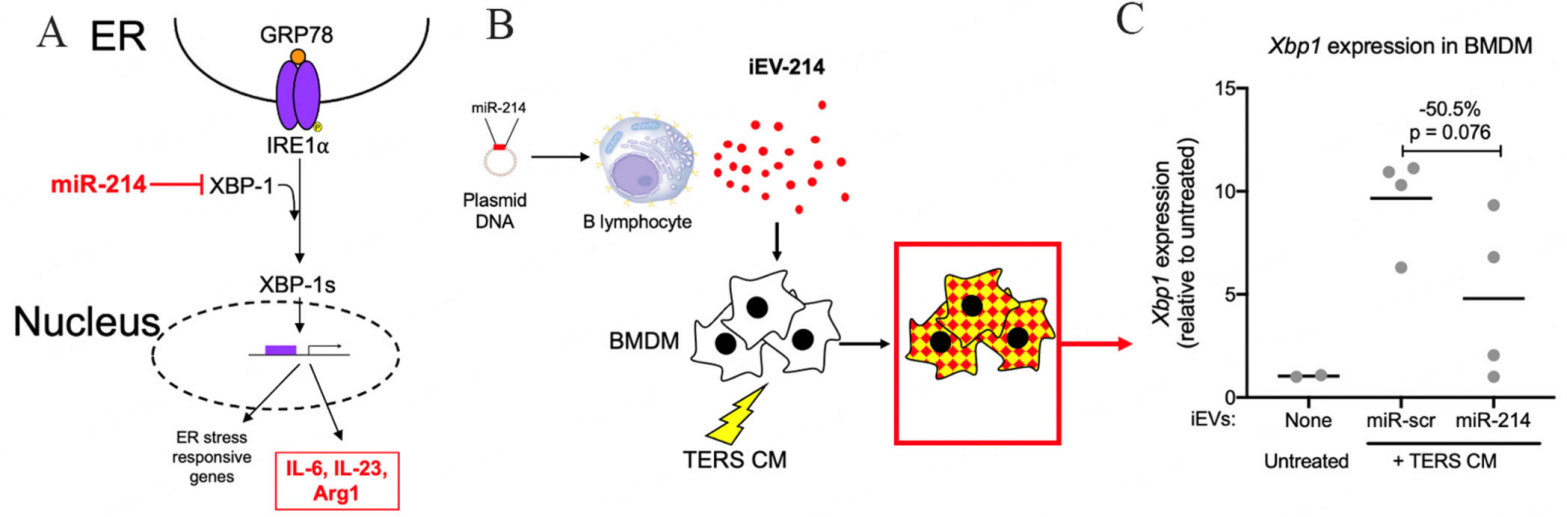
miR-214 downregulates *Xbp1* expression in BMDM. (A) Schematic representation of the IRE1-XBP1 axis and the effects of miR-214; (B) Schematic representation depicting the biological material (BMDM treated with iEVs and TERS CM) analyzed in C; (C) qPCR analysis of *Xbp1* in BMDM that were treated with iEVs and TERS CM. Each data point represents an independent experiment with independently-derived BMDM (biological replicates). Data are shown as fold change relative to untreated conditions and normalized using -actin.

During an ER stress response, canonical stress sensors (IRE1a, PERK, and ATF6) are activated, initiating the UPR. Specifically, IRE1a RNAse activity splices XBP1 mRNA, yielding the biologically active XBP1s [Figure 3A]. Under stress conditions, macrophages undergo the activation of the IRE1-XBP1 axis, and this in turn leads to the promotion of a mixed pro-inflammatory/immune suppressive phenotype^[9]^. Therefore, it became important to determine whether iEV-214 could downregulate Xbp1 expression in conditions of ER stress. In these experiments, stress was induced using TERS CM (see Materials and Methods), which induces a macrophage phenotype that is equivalent to that of macrophages found in the tumor microenvironment of induced and spontaneously arising tumors^[9,10]^.

We treated BMDM with iEV-214 and TERS CM for 24 h and measured the amount of Xbp1 using qPCR [Figure 3B]. TERS CM induced Xbp1 transcription by an average of ~10-fold in the presence of control iEVs (iEV-scr). In contrast, treatment with iEV-214 caused > 50% reduction in Xbp1 transcription, demonstrating that miR-214 delivered via B cell-derived iEVs can effectively downregulate its target mRNA [Figure 3C].

**Figure 4.**
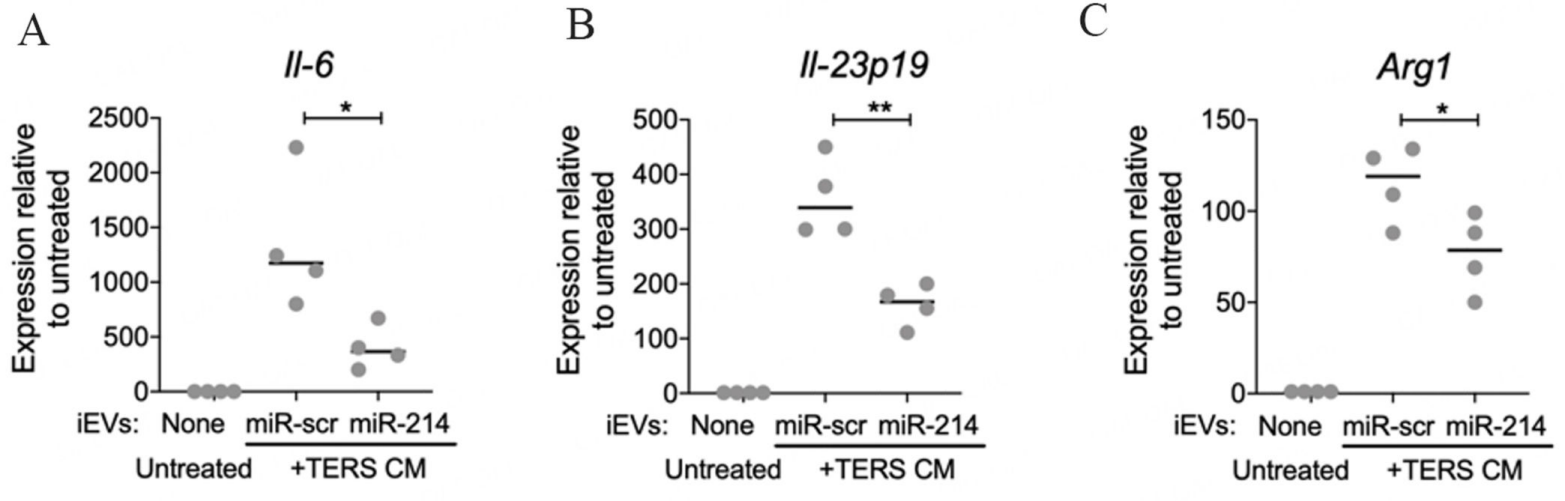
miR-214 reduces the IIS signature in BMDM. qPCR analysis of (A) *Il-6* (B) *Il-23p19* (C) *Arg1* in BMDM that were treated with iEVs and TERS CM. Each data point represents an independent experiment with independently-derived BMDM (biological replicates). Data are shown as fold change relative to untreated conditions and normalized using -actin. *P < 0.05 **P < 0.01.

BMDM activated by TERS CM and treated with iEV-214 had significantly reduced expression of the proinflammatory/pro-tumorigenic cytokine *Il-6* [Figure 4A] and *Il-23p19* [Figure 4B] genes relative to controls. The immune-suppressive gene *Arg1* was also significantly suppressed [Figure 4C]. The transcription of each gene was dramatically induced by TERS CM, and treatment with iEV-214 significantly reduced their induction. This result indicates that iEV-214 not only downregulate target mRNA (*Xbp1*), but also affect downstream genes of pathophysiological relevance in macrophages in the context of cancer.

## DISCUSSION

In the TME, macrophages act as tumor-promoting cells through complex functions that have been classically ascribed to their M1- or M2-type phenotype^[24]^. However, mounting evidence suggests that TME-associated macrophages have a mixed phenotype that allows them to secrete pro-inflammatory cytokines and immunosuppressive molecules^[9,25,26]^, a phenomenon also documented in humans^[27]^. Recently, we reported that the acquisition of this phenotype is under the control of the IRE1-XBP1 axis^[10]^, making Xbp1 an important target in macrophages within the TME. As a proof-of-principle here, we targeted Xbp1 in BMDM using iEVs engineered to carry miR-214, a short non-coding RNA complementary to the 3’-UTR of Xbp1^[22]^.

Engineered miR-214-laden iEVs expressing high levels of miR-214 were readily internalized by BMDM, efficiently transferring their miR-214 cargo. Surprisingly, we found the endogenous levels of mir-214 in these cells to be below the detection level of our assay. An important effect of iEVs internalization and miR-214 transfer was the downregulation of the target mRNA. Consistently, we found > 50% reduction in Xbp1 transcriptions after 24 h of treatment, demonstrating that miR-214 downregulates Xbp1 in macrophages in addition to cardiomyocytes^[21]^ and hepatocellular cancer cells^[22]^, implying that Xbp1 negative regulation by exogenous miR-214 is not cell type-restricted.

In the TME, macrophages display a mixed phenotype characterized by the upregulation of pro-inflammatory cytokines such as IL-6^[28]^ and IL-23^[29]^, but also the immune suppressive factor Arginase 1, which restricts T cell function^[30]^. This phenotype has been found in cancer patients^[27]^ and has been modeled *in vitro* by treating BMDM with TERS CM^[9,10]^. Since the induction of this mixed pro-inflammatory/immune suppressive phenotype is Xbp1-dependent, it was important to document that miR-214-laden iEVs also negatively affect these downstream genes during conditions of stress activation.

The miR-214 effects reported here relied on iEVs as delivery vehicles. iEVs are extracellular vesicles with an average diameter of 100 nm generated in B cells^[23]^. Therefore, iEVs are readily internalized by macrophages via phagocytosis^[31]^ even in the absence of specific targeting molecules (e.g., an antibody or mannose). Delivery of therapeutic iEVs targeting macrophages can leverage macrophage phagocytic activity in a number of human cancer types either by direct intra-tumoral injection or by intra-cavity injection, e.g., peritoneum in ovarian cancer and brain cavity after glioblastoma multiforme removal. iEVs protect the miR cargo from degradation by intracellular enzymes, allowing their cargo miR to have long-lasting effects within the cell and tissue, as recently demonstrated in a model of triple-negative breast cancer where cancer cells were targeted ex vivo with iEVs to restore the intracellular content of a tumor suppressive miR^[23]^.

The limitation of the present study is that all experiments have been performed *in vitro* using optimized experimental conditions. Although the results clearly demonstrate the benefit of mir-214-laden iEVs to treat stressed BMDM and significantly reduce the primary mRNA target as well as relevant co-regulated pro-inflammatory and immune suppressive genes, the benefit *in vivo* can only be extrapolated. In addition, the extent to which miR-214 affect additional genes and gene networks, and the implication of such offtarget effects will need to be systematically assessed through RNASeq. However, as a new principle, the approach reported herein may be applicable to clinical situations in which patients develop resistance to chemotherapy (e.g., platinum and temozolomide resistance) or when the use of new forms of immunotherapy is anticipated to fail due to tumor-infiltrating macrophages. miRNA-laden iEVs targeting macrophages may offer advantages for which a risk-benefit trade-off will need to be assessed on a case-by-case basis.

## CONCLUSIONS

Here, we show that it is possible to target the transcription factor *Xbp1* in macrophages and have a direct impact on the transcription of its mRNA and additionally on the regulation of downstream genes that collectively support the TME macrophages’ pro-tumorigenic role. *In vivo* efficacy studies in preclinical tumor models in which macrophages are known to play a pathogenetic role (e.g., ovarian, breast, glioblastoma) are therefore warranted. These findings suggest a general approach to reprogramming macrophages in disease.

## Data Availability

All data are shown in the paper, and pCMV-MIR-214 is available upon request. All other reagents are available commercially.

## References

[1] MoriK. Signalling pathways in the unfolded protein response: development from yeast to mammals. J Biochem 2009;146:743–50. DOI19861400 10.1093/jb/mvp166

[2] WalterP, RonD. The unfolded protein response: from stress pathway to homeostatic regulation. Science 2011;334:1081–6. DOI22116877 10.1126/science.1209038

[3] LeeAH, IwakoshiNN, GlimcherLH. XBP-1 regulates a subset of endoplasmic reticulum resident chaperone genes in the unfolded protein response. Mol Cell Biol 2003;23:7448–59. DOI PMC14559994 10.1128/MCB.23.21.7448-7459.2003PMC207643

[4] ToddDJ, McHeyzer-WilliamsLJ, KowalC, XBP1 governs late events in plasma cell differentiation and is not required for antigen-specific memory B cell development. J Exp Med 2009;206:2151–9. DOI PMC19752183 10.1084/jem.20090738PMC2757870

[5] KamimuraD, BevanMJ. Endoplasmic reticulum stress regulator XBP-1 contributes to effector CD8+ T cell differentiation during acute infection. J Immunol 2008;181:5433–41. DOI PMC18832700 10.4049/jimmunol.181.8.5433PMC2776092

[6] MahadevanNR, AnufreichikV, RodvoldJJ, ChiuKT, SepulvedaH, ZanettiM. Cell-extrinsic effects of tumor ER stress imprint myeloid dendritic cells and impair CD8+ T cell priming. PLoS One 2012;7:e51845. DOI PMC23272178 10.1371/journal.pone.0051845PMC3525659

[7] IwakoshiNN, PypaertM, GlimcherLH. The transcription factor XBP-1 is essential for the development and survival of dendritic cells. J Exp Med 2007;204:2267–75. DOI PMC17875675 10.1084/jem.20070525PMC2118458

[8] ChopraS, GiovanelliP, Alvarado-VazquezPA, IRE1α-XBP1 signaling in leukocytes controls prostaglandin biosynthesis and pain. Science 2019;365:eaau6499. DOI10.1126/science.aau649931320508

[9] MahadevanNR, RodvoldJ, SepulvedaH, RossiS, DrewAF, ZanettiM. Transmission of endoplasmic reticulum stress and proinflammation from tumor cells to myeloid cells. Proc Natl Acad Sci U S A 2011;108:6561–6. DOI PMC21464300 10.1073/pnas.1008942108PMC3081038

[10] BatistaA, RodvoldJJ, XianS, IRE1α regulates macrophage polarization, PD-L1 expression, and tumor survival. PLoS Biol 2020;18:e3000687. DOI PMC10.1371/journal.pbio.3000687PMC730779432520957

[11] MartinonF, ChenX, LeeAH, GlimcherLH. TLR activation of the transcription factor XBP1 regulates innate immune responses in macrophages. Nat Immunol 2010;11:411–8. DOI PMC20351694 10.1038/ni.1857PMC3113706

[12] ZanettiM, RodvoldJJ, MahadevanNR. The evolving paradigm of cell-nonautonomous UPR-based regulation of immunity by cancer cells. Oncogene 2016;35:269–78. DOI25893303 10.1038/onc.2015.108

[13] BartelDP. MicroRNAs: genomics, biogenesis, mechanism, and function. Cell 2004;116:281–97. DOI14744438 10.1016/s0092-8674(04)00045-5

[14] AmbrosV. The functions of animal microRNAs. Nature 2004;431:350–5. DOI15372042 10.1038/nature02871

[15] PedersenI, DavidM. MicroRNAs in the immune response. Cytokine 2008;43:391–4. DOI PMC18701320 10.1016/j.cyto.2008.07.016PMC3642994

[16] O’ConnellRM, ChaudhuriAA, RaoDS, BaltimoreD. Inositol phosphatase SHIP1 is a primary target of miR-155. Proc Natl Acad Sci U S A 2009;106:7113–8. DOI PMC19359473 10.1073/pnas.0902636106PMC2678424

[17] VoliniaS, CalinGA, LiuCG, A microRNA expression signature of human solid tumors defines cancer gene targets. Proc Natl Acad Sci U S A 2006;103:2257–61. DOI PMC16461460 10.1073/pnas.0510565103PMC1413718

[18] AlmanzaG, AnufreichikV, RodvoldJJ, Synthesis and delivery of short, noncoding RNA by B lymphocytes. Proc Natl Acad Sci U S A 2013;110:20182–7. DOI PMC24277816 10.1073/pnas.1311145110PMC3864290

[19] AlmanzaG, ZanettiM. High-efficiency generation of multiple short noncoding RNA in B-cells and B-cell-derived extracellular vesicles. Mol Ther Nucleic Acids 2015;4:e271. DOI PMC26670278 10.1038/mtna.2015.44PMC5014536

[20] DuanQ, YangL, GongW, MicroRNA-214 is upregulated in heart failure patients and suppresses XBP1-mediated endothelial cells angiogenesis. J Cell Physiol 2015;230:1964–73. DOI PMC25656649 10.1002/jcp.24942PMC4911176

[21] DuanQ, ChenC, YangL, MicroRNA regulation of unfolded protein response transcription factor XBP1 in the progression of cardiac hypertrophy and heart failure in vivo. J Transl Med 2015;13:363. DOI PMC26572862 10.1186/s12967-015-0725-4PMC4647486

[22] DuanQ, WangX, GongW, ER stress negatively modulates the expression of the miR-199a/214 cluster to regulates tumor survival and progression in human hepatocellular cancer. PLoS One 2012;7:e31518. DOI PMC22359598 10.1371/journal.pone.0031518PMC3281082

[23] AlmanzaG, RodvoldJJ, TsuiB, JepsenK, CarterH, ZanettiM. Extracellular vesicles produced in B cells deliver tumor suppressor miR-335 to breast cancer cells disrupting oncogenic programming in vitro and in vivo. Sci Rep 2018;8:17581. DOI PMC30514916 10.1038/s41598-018-35968-2PMC6279829

[24] MantovaniA, SozzaniS, LocatiM, AllavenaP, SicaA. Macrophage polarization: tumor-associated macrophages as a paradigm for polarized M2 mononuclear phagocytes. Trends Immunol 2002;23:549–55. DOI12401408 10.1016/s1471-4906(02)02302-5

[25] Van GinderachterJA, MovahediK, Hassanzadeh GhassabehG, Classical and alternative activation of mononuclear phagocytes: picking the best of both worlds for tumor promotion. Immunobiology 2006;211:487–501. DOI16920488 10.1016/j.imbio.2006.06.002

[26] Ostrand-RosenbergS, SinhaP. Myeloid-derived suppressor cells: linking inflammation and cancer. J Immunol 2009;182:4499–506. DOI PMC19342621 10.4049/jimmunol.0802740PMC2810498

[27] ChittezhathM, DhillonMK, LimJY, Molecular profiling reveals a tumor-promoting phenotype of monocytes and macrophages in human cancer progression. Immunity 2014;41:815–29. DOI25453823 10.1016/j.immuni.2014.09.014

[28] KimS, TakahashiH, LinWW, Carcinoma-produced factors activate myeloid cells through TLR2 to stimulate metastasis. Nature 2009;457:102–6. DOI PMC19122641 10.1038/nature07623PMC2746432

[29] LangowskiJL, ZhangX, WuL, MattsonJD, ChenT, SmithK, BashamB, McClanahanT, KasteleinRA, OftM. IL-23 promotes tumour incidence and growth. Nature 2006;442:461–5. DOI16688182 10.1038/nature04808

[30] NorianLA, RodriguezPC, O’MaraLA, Tumor-infiltrating regulatory dendritic cells inhibit CD8+ T cell function via L-arginine metabolism. Cancer Res 2009;69:3086–94. DOI PMC19293186 10.1158/0008-5472.CAN-08-2826PMC2848068

[31] FengD, ZhaoWL, YeYY, Cellular internalization of exosomes occurs through phagocytosis. Traffic 2010;11:675–87. DOI20136776 10.1111/j.1600-0854.2010.01041.x

